# Application of plan-do-check-act management to improve first-attempt insertion success rates of internal jugular vein catheterization for standardized training residents in an intensive care unit

**DOI:** 10.1186/s12909-022-03418-3

**Published:** 2022-06-02

**Authors:** Fang Lai, Dongping Xie, Yanna Weng, Shutao Mai, Jiongdong Du, Yun Han, Yan Zhang

**Affiliations:** 1 The Second Affiliated Hospital of Guangzhou University of Chinese Medicine, Guangzhou, Guangdong China; 2 Guangdong Provincial Hospital of Chinese Medicine, Guangzhou, Guangdong China; 3 Chao En-xiang Famous Chinese Medicine Expert Inheritance Studio, Guangzhou, Guangdong China

**Keywords:** Plan-do-check-act cycle, Standardized training residents, Internal jugular vein catheterization

## Abstract

**Background:**

In the intensive care unit (ICU), internal jugular vein puncture and catheterization are basic rescue operations that physicians need to complete quickly and independently. It is necessary to improve the first-attempt success rate of internal jugular vein catheterization, shorten the catheterization duration and reduce the incidence of complications for standardized training residents (STRs).

**Objective:**

To improve first-attempt insertion success rates of internal jugular vein catheterization for STRs.

**Methods:**

Based on the PDCA cycle management method and current situation investigation, the PDCA management objectives were set, and the implementation content, monitoring items and continuous improvement plan were formulated. The data of residents who were trained in the ICU of Fangcun Hospital, Second Affiliated Hospital of Guangzhou University of Chinese Medicine, from January 2016 to April 2016 and managed by the PDCA cycle (PDCA group), were compared with the data of residents trained in the same department from August 2015 to November 2015 before the implementation of PDCA (historic control group), the first-attempt success rate of puncture and catheterization, the duration of puncture and catheterization, and the incidence of complications were analysed.

**Results:**

Thirty-six cases of internal jugular vein catheterization were performed by the PDCA group, 21 cases (58%) were performed by residents in the third year of standardized training, and 15 cases (42%) were performed by residents in the second year of standardized training. Compared with the historic control group, there was no significant difference in the seniority of residents (*X*^2^ = 0.240, *P* = 0.625) or the ‘majors of the residents (*X*^2^ = 1.306, *P* = 0.835). The first-attempt success rate of puncture in the PDCA group was 94% (34/36), which was significantly higher than that of the historic control group (55% (11/20) (*P* = 0.001). In the PDCA group, the first-attempt success rate of puncture among third-year standardized training residents was 95% (20/21), and the first-attempt success rate in the second-year was 93% (14/15), which were significantly higher than the corresponding rates of 62% (8/13) and 43% (3/7) respectively, in the historic control group (all *P* = 0.021). The duration of catheterization was [4 (3,5)] min after PDCA, which was significantly shorter than that in the historic control group [9 (6.25,13.00)] min (*Z* = − 5.214, *P* < 0.001). The incidence rate of complications in the PDCA group was 0% (0 /36), which was significantly lower than the rate of 20% (4 / 20) in the historic control group (*P* < 0.013).

**Conclusion:**

PDCA cycle management can help improve the first-attempt success rate of internal jugular vein puncture and catheterization, shorten the duration of puncture and catheterization, and reduce the incidence of complications. The idea and method of PDCA cycle management can be applied to other training and management protocols for STRs.

## Introduction

In the intensive care unit (ICU), deep vein catheterization is one of the basic rescue operations that residents need to complete quickly and independently. Internal jugular vein puncture and catheterization may be the first choice for deep venous catheterization mostly because of its advantages of ease of operation, ease of use to compress and stop bleeding, ability to monitor central venous pressure, and low risk of infection in long-term care. However, there are still some risks in internal jugular vein puncture, such as inadvertent arterial puncture, inadvertent pleural cavity puncture, local haematoma, etc. Thus, it is necessary to improve the first-attempt success rate of internal jugular vein catheterization, shorten the catheterization time duration and reduce the incidence of complications for physicians.

With the promotion of standardized training of residents, STRs have become an important part of the front-line clinical staff in the ICU. Improving the quality of the standardized training of residents, not only to promote the steady growth of young residents, but also to ensure the medical safety of critically ill patients, is the focus and difficulty of clinical teaching in the ICU.

The PDCA cycle was a quality management method that divides management into four stages: the first stage is “Plan”, that is, to formulate work objectives; the second stage is “Do (execution)”, that is, to realize the contents in the plan; the third stage is “Check”, which summarizes the results of plan implementation and determines the problem; the fourth stage is “Act (processing)”, that is, the summary of processing inspection, successful experience is incorporated into standardized management, and unsolved problems enter the next PDCA cycle. Through a continuous PDCA cycle, the problems in quality management are gradually solved to realize standardized management [1].

We supposed that the application of plan-do-check-act (PDCA) cycle management could help to improve the first-attempt insertion success rates of internal jugular vein catheterization for standardized training residents (STRs).

By applying the PDCA cycle to the training and quality management of internal jugular vein puncture and catheterization for ICU STRs, we summarized and identified the main reasons for repeated puncture and catheterization failures in our department, put forward corresponding rectification measures for each reason, and significantly improved the first-attempt success rate of internal jugular vein puncture and catheterization for ICU STRs. The duration of catheterization was shortened and the incidence of complications was reduced. The research contents are summarized and reported as follows.

## Methods

### Participants

From January 2016 to April 2016, STRs in ICU of Fangcun Hospital, the Second Affiliated Hospital of Guangzhou University of Chinese medicine (PDCA group) were evaluated.

### PDCA cycle

We used the PDCA cycle to formulate the training and quality control plan. Compared with the data of the STRs trained in the same department from August 2015 to November 2015 (the historic control group), the first-attempt puncture success rates, time duration and complication incidence rates of puncture and catheterization were analysed. Only the first operation of each STR was included for statistical analysis.

#### P——plan

1. Definition
  We clearly defined the “first-attempt success rate”, “puncture and catheterization duration”, “catheteriazation failure” from the start.
  1. First-attempt success rate: successful catheterization with only one insertion in the puncture.
  2. Puncture and catheterization duration: the duration from puncture to catheter fixation.
  3. Catheterization failure: failure of catheterization and the need for puncture in other body parts (such as the subclavian vein or femoral vein).
2. Data collection
  1. Literature survey: We conducted a literature survey to optimize the training program and specify training objectives.
  2. Current situation investigation: From August 2015 to November 2015, we carried out traditional clinical teaching regarding internal jugular vein catheterization. Specifically: we conducted lectures about the indications, contraindications, operation steps and precautions of internal jugular vein catheterization once a month; residents observed the operation of internal jugular vein catheterization by supervisory physicians; with the permission of supervisory physicians, residents could carry out the operation of internal jugular vein catheterization independently; and ultrasound-guided puncture was not needed.
  We calculated the number of cases operated on by STRs, the first-attempt success rate, the duration of puncture and catheterization and the incidence of complications.
3. A fishbone Diagram was used to summarize the possible influencing factors. The PDCA project team was established to analyse the reasons for catheterization that was not successful at the first attempt by combining own experience with literature reports.
4. Develop objectives:
  For the main causes confirmed above, the PDCA project team developed objectives for PDCA management.

#### D——do

1. Formulate standardized operation process
  By referring to the central venous puncture and catheterization section in the “Qualification Training Textbook for Critical Care Medicine Specialty”, we formulated the ultrasound-assisted operation process specification and distributed it to the residents for training. We conducted training through slide presentations, videos and bedside teaching once a month.
2. Implement operation access system
  1. Residents could not carry out clinical practice before passing the qualification assessment. We provided molds upon which residents could practice.
  2. According to the invasive operation access system formulated by the hospital, after training and assessment, residents could participate in the operation as the first assistant. After being the first assistant in up to 5 cases, the supervisory physician assessed the qualification of residents as the main operator by using a unified scoring table. After passing the examination of the supervisory physician, residents could carry out clinical practice as the main operator under the guidance of the supervisory physician.
3. Implement ultrasound assisted puncture
  1. Ultrasound had to be used for vascular evaluation and puncture site location before the operation.
  2. For patients who had an obvious vascular variation that caused difficulty locating the proper puncture point by ultrasonic evaluation before the operation, residents were required to ask the supervisory physician whether to change the position of catheterization. If the catheterization position was not changed, with approval of the supervisory physician, the ultrasound-guided puncture could be conducted by the residents.

#### C——check

1. Monitor the implementation of the standardized operation process: The operation monitoring flow sheet was formulated and then accurately recorded by the supervisor (usually the supervisory doctor or bedside nurse) during the operation.
2. The names of the operators and assistants were required to be accurately recorded. The acquisition of operation access could be reviewed at any time.

#### A——act

1. We summarized the monitoring forms and analysed the reasons affecting the standard implementation and successful catheterization weekly to specify the objectives of next stage management.
2. We summarized the misunderstandings, knowledge-related blind spots and errors of the residents in practice, which would be the focus of training in the next stage.

### Statistical analysis

SPSS 17.0 software was used for statistical analysis. The measurement data conforming to a normal distribution are expressed as the mean ± standard deviation ($\overline{x}\pm s$), the measurement data of skew distribution are expressed as the median and interquartile spacing *M* (*p25-p75*), and the counting data are expressed as the frequency and percentage (*n*,%). The Shapiro-Wilk test was used for normality testing. Levene’s test was used for variance homogeneity (inspection level *α* = 0.10 for the homogeneity test). When the measurement data conformed to normal distribution and homogeneity of variance, *t* test was used for intergroup comparison. A nonparametric test was used for data that did not conform to a normal distribution and homogeneity of variance, and the Mann-Whitney U rank sum test was used for intergroup comparisons. Count data were compared with the *X*^*2*^ test, when the actual frequency was small, Fisher’s exact probability method was used. The inspection level was *α* = 0.05.

## Results

### PDCA cycle

**Fig. 1:**
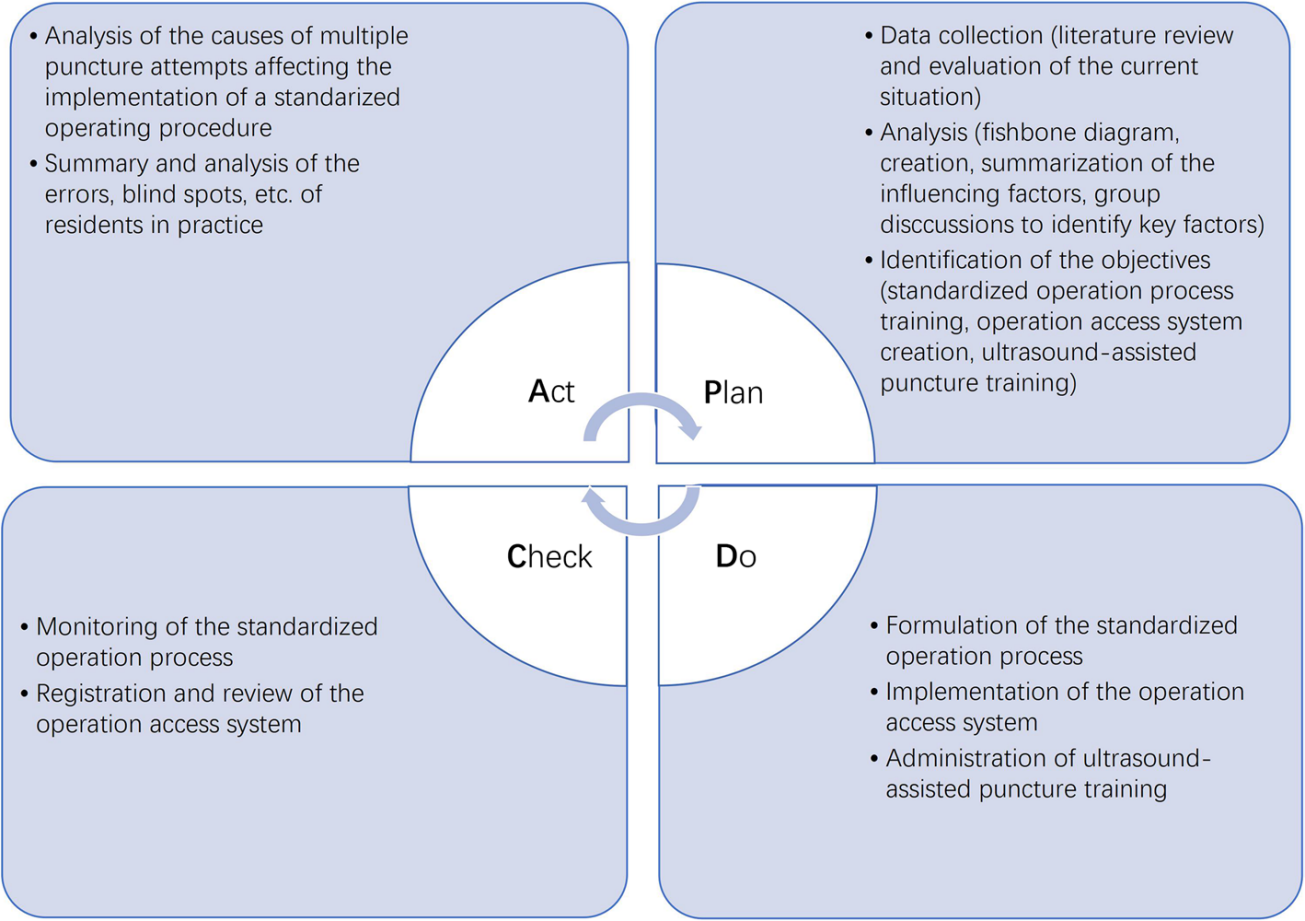
PDCA cycle to improve the first attempt success rates of internal jugular vein puncture and catheterization for STRs

The PDCA cycle was used to formulate the training and quality control plan (Fig. 1).

### The possible influencing factors summarized with fishbone diagrams

**Fig. 2:**
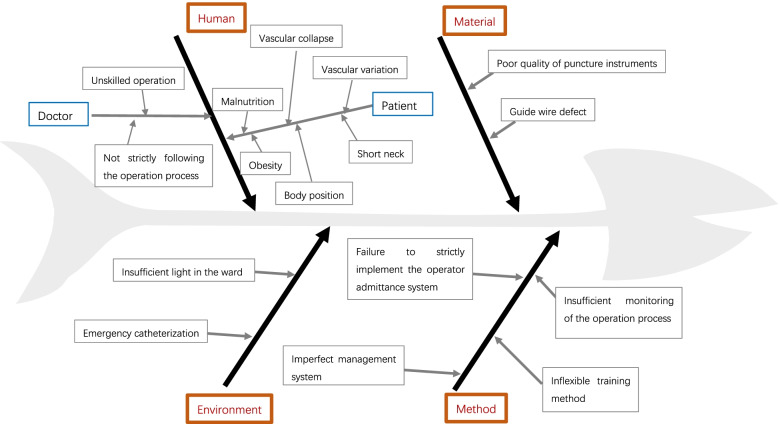
Fishbone diagram of possible influencing factors for repeated puncture or catheterization failure. ① Human. Doctor: unskilled operation, not strictly following the operation process. Patient: obesity, short neck, malnutrition, body position limitation (not being able to lie flat for any reason), vascular variation, vascular collapse, poor cooperation. ② Material:poor quality of puncture instruments, guide wire defect. ③ Environment: insufficient light, emergency catheterization. ④ Method: failure to strictly implement the operator admittance system, imperfect management system, insufficient operation process monitoring, inflexible training method

Through a literature review, we summarized the possible influencing factors with a fishbone diagram (Fig. 2).

### The main causes of multiple puncture attempts in our own department

The PDCA project team discussed each possible influencing factor summarized with a fishbone diagram, and confirmed the main causes in our department (Table 1).

**Table 1:** PDCA project team confirmation of the main causes by group discussion

No.	Possible factor	Actual situation	Main cause confirmation
1	Not strictly following the operation process	Residents’ knowledge of the operation is uneven.	Yes
2	Unskilled operation	Unskilled operation leads to repeated puncture.	Yes
3	Vascular variation	Ultrasound confirmed vascular variation in some patients.	Yes
4	Difficulty in locating the puncture point on the body surface	Patients with obesity, a short neck, malnutrition or severe oedema are prone to undergoing repeat puncture due to difficulty in locating the proper puncture point.	Yes
5	Vascular collapse	Ultrasound-confirmed extreme vascular collapse is rare.	No
6	Poor cooperation of patients	Patients cooperate well after sedation and analgesia.	No
7	Poor quality of puncture instruments	The first-attempt success rate of skilled operators is high with the same model of puncture instrument.	No
8	Insufficient light in the ward	Insufficient light in the ward is rare.	No
9	Emergency catheterization	Emergency catheterization will always be conducted by skilled supervisory physicians.	No

The reasons for catheterization that could not be successful at one puncture attempt in our department were as follows: ① not strictly follow the operation process; ② unskilled operation; ③ vascular variation; and ④ difficult in locating the puncture point on the body surface.

### Objectives of PDCA management

For the main causes confirmed above, the PDCA project team developed the objectives of PDCA management:

1. 100% standardized training rate: all residents should undergo standardized operation training.
2. 100% access qualification acquisition rate: strictly implement the operator admittance system. The operators should all gain admittance before performing internal jugular vein puncture and catheterization on patients in our department.
3. The success rate of the first attempt of internal jugular vein puncture and catheterization was 95%. We trained residents to use ultrasound to improve the success rate of the first attempt of internal jugular vein puncture and catheterization. The literature reports that the first-attempt success rate of ultrasound-guided puncture and catheterization can be as high as 95% [2, 3].

### Baseline of operators

In the PDCA group, the residents underwent internal jugular vein puncture and catheterization for 36 cases. Twenty-one cases (58%) were implemented by residents in the third year of the standardized training, and 15 cases (42%) were in the second year. Compared with the historic control group, there was no significant difference in the seniority of residents and the majors of the residents. The specific results are shown in Table 2.

**Table 2:** Baseline of the operators

	historic control group	PDCA group	*X*^2^	*P* value
Number of cases	20	36	0.240	0.625
Residents in the third year of standardized training [cases, n(%)]	13(65%)	21(58%)		
Residents in the second year of standardized training [cases, n(%)]	7(35%)	15(42%)		
Majors of the residents			1.3060^a^	0.835
Internal medicine	9 (45%)	17 (47%)		
Anaesthesiology	1 (5%)	1 (3%)		
Surgery	3 (15%)	3 (8%)		
Undetermined	7 (35%)	15 (42%)		

^a^: Fisher exact probability method

### Comparison of first-attempt puncture success rates

The first-attempt puncture success rate of the PDCA group was 94% (34/ 36), which was significantly higher than that of the historic control group. In the PDCA group, the first-attempt puncture success rate for the operators in the third year of standardized training was 95%, and the first-attempt puncture success rate for the operators in the second year was 93%, which was significantly higher than the corresponding rates in the historic control group. See Table 3 for details.

**Table 3:** Comparison of first-attempt puncture success rates

	historic control group	PDCA group	*X*^2^	*P* value
Number of first-attempt puncture success cases [cases, n (%)]	11(55%)	34(94%)	—^a^	0.001
Residents in the third year of standardized training [cases, n (%)]	8(62%)	20(95%)	—^a^	0.021
Residents in the second year of standardized training [cases, n (%)]	3(43%)	14(93%)	—^a^	0.021

^a^Fisher exact probability method

### Comparison of the duration of catheterization and incidence rates of complications

The duration of catheterization was [4 (3,5)] min in the PDCA group, which was significantly shorter than that in the historic control group.

The incidence rate of complications of the PDCA group was 0%, which was significantly lower than that in the historic control group. See Table 4 for details.

**Table 4:** Comparison of the duration of catheterization and incidence rates of complications

	historic control group	PDCA group	*Z*	*P* value
Duration of catheterization [min, M (Q25, Q75)]	9(6.25, 13.00)	4(3, 5)	*Z* = -5.214	0.000
Incidence of complications [cases, n (%)]	4(20%)	0(0%)	—^a^	0.013
Inadvertent arterial puncture	1(5%)	0(0%)		
Bleeding or haematoma	3(15%)	0(0%)		
Pneumothorax, haemothorax or chylothorax	0(0%)	0(0%)		
Catheter-associated infection	0(0%)	0(0%)		

^a^: Fisher exact probability method

## Discussion

### PDCA can help improve internal jugular vein catheterization training of STRs in the ICU

In China, residents begin to undergo standardized training after obtaining a bachelor’s degree. Most of the residents obtain their medical practitioner certificate and are registered in the second or the third year of training. No invasive operation can be carried out by residents without the qualification of medical practitioner. Therefore, most STRs begin to have experience as main operator of deep vein catheterization in their second or third year of standardized training. The ICU is one of the main sites for implementation of deep venous catheterization. When residents rotate through training in the ICU, they often have insufficient relevant knowledge and practical experience. However, ICU patients are often in a poor comprehensive state and critical condition, which imposes high requirements on operators.

In this study, the PDCA circulation management method was adopted, and the literature and current situation investigation were carried out. A literature survey revealed that the first-attempt success rates of internal jugular vein catheterization by the traditional standard body surface positioning method were 58.3–78.4% [4], while the first-attempt success rate of ultrasound-guided puncture and catheterization could be as high as 95% [2, 3], the average puncture and catheterization duration was shortened to 25% [2], and the incidence rates of arterial puncture decreased from 8.3–12% to 0–1.7% [2, 3, 5]. There was a negative correlation between the operator’s proficiency and the incidence of operation-related mechanical injury [5]. Mechanical complications (such as accidental puncture of an artery, haematoma, haemothorax and pneumothorax) will increase more than 6 times with more than 3 attempts of failed punctures [6, 7]. The results of this study are consistent with the literature reports.

This study revealed that, after standardized and effective training, junior training residents can also quickly and safely complete basic procedures such as internal jugular vein catheterization, which can increase their sense of achievement and self-confidence. At the same time, it can spare supervisory physicians more time and energy to deal with more complex clinical problems, which is helpful to optimize the clinical processing ability of the medical team. Therefore, after PDCA circulation management, the number of cases of internal jugular vein puncture and catheterization operated by STRs and the proportion of junior STR operators have increased. In addition, detailed training and quality control requirements have also helped to cultivate a rigorous work style for the residents.

### Appropriate puncture approach and standard operating procedures compliance are keys to successful catheterizations

In recent years, ultrasound has developed rapidly in critical medicine. Bedside ultrasound machines are easily available in many medical institutions. The use of ultrasound in vascular puncture and catheterization is the most basic application. Many studies have reported that ultrasound-guided central venous catheterization can improve the success rate of puncture, reduce the length of puncture duration and reduce the incidence of complications related to puncture and catheterization [8, 9]. However, puncture and catheterization with ultrasound guidance does not mean that the approach can be selected casually. Even with ultrasound guidance, an inappropriate puncture approach may also cause serious damage to nerves and muscles [10, 11]. Thus, it is very important to follow the classical approach for puncture. Furthermore, even if guided by ultrasound, the complications related to catheterization by unskilled operators are much higher than those related to catheterization by skilled operators [12]. Failure to follow the procedures may result in serious adverse consequences. For example, the literature has reported cases of a catheter guide wire being left in the patient’s body after catheterization [13, 14]. 

Therefore, the application of ultrasound may bring benefits to central venous catheterization, which is based on the operator’s full mastery of anatomical structure, operating procedures, correct ultrasound use and other relevant knowledge.

As a result, we did not require trained residents to operate under the guidance of real-time ultrasound, but only required the use of ultrasound to evaluate the target puncture vessel before the operation, which yielded good results. Therefore, for most of the standardized training residents, it is not necessary to use the ultrasonic device specially for guiding puncture, or to be very familiar with the operation skills of puncture under the real-time positioning of ultrasound. However, it is very important for residents to master the basic skills of puncture vessel evaluation with ultrasound before operation if bedside ultrasound is available. Based on the ultrasound evaluation before the operation, an appropriate approach is selected. For difficult cases, asking a supervisory doctor or a more experienced doctor for help in time can avoid inevitable failure.

### Goal orientation is important not only in PDCA management but also in clinical teaching

Scientific and correct goal setting is an important factor to achieve good results in PDCA cycle management. In this study, we required the STRs to achieve the operation principles of “accurate, stable and fast”, and the order of the three words could not be reversed. First, we should avoid repeated puncture as much as possible. As time permits, it is better to spend sufficient time clarifying the vascular anatomy and making enough preparations. Second, the operation needs to comply with the procedural specifications, and residents must pay attention to various details. To be fast enough is the last requirement. The duration assessment in this study was the duration of puncture and catheterization (from the beginning of puncture to fixing the catheter). In other studies, it is often reported as the puncture time duration (from needle insertion to venous blood extraction). Therefore, the duration reported in those studies is often as short as a few seconds. The reason for this goal setting is that we hope our residents will regard “smoothly indwelling a venous channel” as the objective, rather than “successfully penetrating the vein”.

### Limitations

The main limitation of this study is related to its inherent bias. In the PDCA group, all residents were required to perform ultrasound scans before operation. Difficult catheterization would be submitted to a superior for further processing. This might be an important factor to ensure the success of residents’ first puncture attempts.

Futhermore, the conclusions of this study are based on only a single center, and compared with historical data, the sample size is relatively small. Therefore, we should pay attention to these limits when considering this report.

## Conclusion

PDCA circulation management could help to improve the first-attempt success rate of internal jugular vein puncture and catheterization for standardized training residents in the ICU, shorten the puncture and catheterization duration and reduce the incidence of complications. The method of PDCA cycle management could be applied to training and management for various skills of residents and specialists.

## Data Availability

The data used and/or analysed during the current study are available from the corresponding author on reasonable request.

## Abbreviations

ICU: Intensive care unit
STR: Standardized training resident
